# Online Vape Shop Customers Who Use E-Cigarettes Report Abstinence from Smoking and Improved Quality of Life, But a Substantial Minority Still Have Vaping-Related Health Concerns

**DOI:** 10.3390/ijerph14070798

**Published:** 2017-07-17

**Authors:** Dinska Van Gucht, Karolien Adriaens, Frank Baeyens

**Affiliations:** 1 Applied Psychology Unit, Thomas More Mechelen-Antwerp University College, Molenstraat 8, 2018 Antwerp, Belgium; 2 Faculty of Psychology and Educational Sciences, KU Leuven—University of Leuven, Tiensestraat 102, 3000 Leuven, Belgium; karolien.adriaens@kuleuven.be (K.A.); frank.baeyens@kuleuven.be (F.B.)

**Keywords:** electronic cigarette, online vape shop customers, vaping, smoking cessation, perception

## Abstract

(1) Background: Characteristics and usage patterns of vapers (e-cigarette users) have mainly been studied in web-based convenience samples or in visitors of brick-and-mortar vape shops. We extended this by targeting customers of one particular online vape shop in the Netherlands; (2) Methods: Customers were questioned on their smoking history, current smoking and vaping status, reasons for vaping, perceived harmfulness, and potential health changes due to vaping; (3) Results: Almost everyone (99%, 95% CI 0.96, 1.00) smoked before they started vaping. A great majority agreed that unlike with other smoking-cessation aids, they could quit smoking (81%, 95% CI 0.79, 0.90) due to vaping. Almost all customers were regular vapers (93.6%, 95% CI 0.89, 0.96) who used state-of-the-art open system devices without modifications and e-liquid with 10 mg/mL nicotine on average. Vapers reported using e-cigs to quit smoking, because e-cigs are healthier, and for financial reasons. The majority (52.6%, 95% CI 0.46, 0.60) perceived vaping as *not that harmful* to *not harmful at all*, but one fifth (21.8%, 95% CI 0.16, 0.28) believed vaping to be harmful. More than half (57.8%, 95% CI 0.50, 0.65) reported gaining more pleasure from vaping than from smoking. A substantial majority (84.2%, 95% CI 0.78, 0.89) agreed that their health had improved since they started vaping; (4) Conclusions: Findings are similar to those obtained in other vape shop studies, but also to the results of convenience samples of less-well-defined populations.

## 1. Introduction

This study aimed to provide an answer to the question: *What is the typical profile of the (Dutch or Flemish) online vape shop customer*? Quite a few studies have been conducted on the characteristics of vapers and on their e-cig-related patterns of use, preferences, perceptions, and beliefs, mostly using convenience sampling of vapers visiting e-cig or smoking cessation websites and discussion fora, or relying on social media snowball sampling (a sampling method where a link to a survey is posted online and where visitors of the specific website and participants can repost the link on other websites in order to collect more participants) [1,2,3,4]. These web-based convenience sample surveys have provided useful and often converging information about (self-selected) vapers’ behavioral and attitudinal profiles. It is less clear, however, what target population is represented by those samples, and to what extent those convenience samples represent biased sampling of that target population. It is probably safe to assume that there is at least a selection bias for being active on internet and/or social media, and most probably also for having positive experiences with and being enthusiastic about vaping. Even allowing biased sampling, due to the lack of an identifiable target population it is not clear which—if any—of the findings of these surveys can be extrapolated to different populations of vapers, or even to most or all vapers [5]. Thus, we agree with Phillips [5] that “*most results of those surveys can only be said to be descriptions of the sample, rather than any measure of some population’s characteristics*”.

A slightly different approach was followed in two survey studies that focused on customers of brick-and-mortar vape shops; even though still being based on convenience sampling and most probably also selecting on vaping enthusiasm to some degree, at least a better-defined population of a geographically defined group of actual e-cig customers is targeted here. Volesky and colleagues [6] surveyed (Canadian) customers of vape shops in the Ottawa area (those making a purchase in the shop were invited both by means of a flier and via a link to the survey on the company’s Facebook web-page), whereas Tackett and colleagues [7] surveyed customers of Midwestern United States vape stores (all customers entering the vape shops were approached and invited to participate by a research assistant, and about 90% of individuals approached agreed to participate). Central findings in both studies were that nearly all vapers were (long-term, pack-a-day) former (or current) smokers; that most had started vaping as a means to quit or reduce tobacco smoking and experienced vaping as an effective way to do so; that e-cigarettes were experienced by a large majority as helping to improve health and were seen as (relatively) harmless; that almost all customers vaped nicotine-containing e-liquids using state-of-the-art advanced open system e-cigs; and that many enjoyed a choice of flavors and/or non-tobacco/non-menthol flavors and used their e-cig at multiple different places both inside and outside.

The aim of the current study was to extend the survey of brick-and-mortar vape shop customers to those making purchases at an online vape shop. It is an open question whether online vape shops attract similar vapers as brick-and-mortar shops do or, alternatively, cater to different vaping subpopulations (e.g., beginners and dual users vs. dedicated vapers), with different smoking and vaping profiles. The targeted population consisted primarily of adult Dutch vapers (and, secondarily, of Flemish cross-border customers—see Discussion) making online purchases at one particular vape shop based in the Netherlands. According to the Special Eurobarometer 429 [8] (data collected about 12 months before the data collection of the current study), out of the 14 million Dutch over 15 years old, 23% were current smokers, and 10% of the population had tried an e-cig. A total of 2% of the general population were current e-cig users (which translates to about 280,000 Dutch current vapers), whereas current e-cig use in current smokers was estimated at 7%, in ex-smokers at 1%, and in never-smokers at 0%. In Wave 8 of the ITC-Netherlands Survey [9] (data collected 18 months before our study), current use of e-cigs in current smokers was estimated substantially higher at 16%. Sales of e-cigs were regulated by the “Temporary Decree Electronic cigarettes” (“Tijdelijk warenbesluit electronische sigaret”, 24 November 2014 [10])—a precursor of the implementation of the Tobacco Products Directive 2014/40/EU into Dutch law (already including maximum content of e-liquid bottles of 10 mL, maximum content of clearomizers of 2 mL, maximum nicotine concentration in e-liquid of 20 mg/mL, leak-free filling, health warnings on the packaging, but not yet a ban on promotion and advertising nor a ban on sales to minors).

At the time of the study, the products on offer at the online shop mostly catered to the “intermediate-level” vaper—that is, it did not contain any prefilled closed-system or disposable e-cigs, nor did it offer high-end vaping gear or materials for DIY (coil/e-liquid making). So, it was reasonable to assume that the sample surveyed similarly represented a population of regular, open-system vapers (rather than, for example, vape aficionados or novice closed-system users) living in The Netherlands (or Flanders). The sample surveyed consisted of the minority of customers who accepted the invitation to participate, so a certain degree of selection bias based on positive experiences with and enthusiasm about vaping was inevitable (see Methods section). In this first assessment of people placing an order at an online vape shop, the goal was (1) to get a clear picture of these customers’ smoking history and status; (2) to look into their current vaping profile and reasons for vaping; (3) to investigate how these customers perceive the harmfulness of smoking versus vaping, using medically approved nicotine-replacement therapy (NRT), or using quit-smoking medication; and finally (4) to question whether or not customers experienced (health) benefits which they attributed to (having switched to) vaping.

## 2. Materials and Methods

### 2.1. Participants

To study a convenience sample of online vape shop customers, we contacted a representative medium-size (at the time of the survey, there were about 23,000 different registered “ever” online customers) dedicated web shop in The Netherlands (www.e-cig4u.nl). We asked the owner to refer all customers who made an online purchase in the time window 18 December 2015–12 January 2016 on a voluntary basis to our online questionnaire. In less than one month, the web shop had 1311 customers, of which 210 started filling out our questionnaire (response rate 16%). Seven participants did not complete the questionnaire, so a total sample of 203 remained, with more men than women (see Table 1 for more sociodemographic details). The majority had Dutch nationality, about a quarter was Belgian, and a few customers had another nationality.

Customers of the web shop had to be at least 18 years, but there was no formal age verification system to access the website or to place online orders. Those who completed the questionnaire were on average 46 years old, the youngest being 19 and the oldest being 78 years old.

### 2.2. Measures

The online questionnaire was made in Qualtrics and was based on earlier work by Tackett and colleagues [7]. It started with some questions assessing background information: age, gender, nationality, income (open-ended questions) and education and profession (both predefined categories, see above). In a second part, participants were questioned on their smoking history: how old they were when they started smoking, how long they had been smoking, if they had undertaken any quit attempts and if so how many, and which smoking cessation aids (*nicotine patches, nicotine gum, nicotine tablets, inhaler, mouth spray, smoking cessation medication, will power, tobacco counselor, e-cig*) they had used and which had been successful. Current smokers also answered questions on how many cigarettes they were smoking and on their motivation to quit. Those currently smoking also filled out the questions of the Fagerström Test for Cigarette Dependence (FTCD) [11] and rated a list of possible complaints they experienced due to smoking (such as throat aches, headaches, bad smell, etc.—see Table 3 for the full list) on a scale from 1 (never) over 3 (sometimes) to 5 (always).

In a final part, all participants were questioned on their current vaping status. First, we asked for the reasons why participants were using e-cigs (*to quit smoking, to smoke less, dual use, out of curiosity, to pass time, because smoking is prohibited in certain places, different flavors, others do it, financial reasons*). Next, they were asked how long they already had been using them, how much e-liquid they currently consumed on an average week, how many times per day they used their e-cig, and how many puffs they took per vaping bout. We also asked about their favorite flavors and the brand and type of e-cig they currently used. Participants were then asked about the experienced disadvantages of both vaping and smoking (open-ended questions) and to indicate what they perceived as most harmful to their health (“*tobacco cigarettes*”, “*e-cigs*”, “*they are both equally harmful to my health*”, or “*they are both not harmful to my health*”). Subsequently, they were asked to rate the harmfulness to their health of four types of products (*tobacco cigarettes, e-cigs, smoking cessation medications, and nicotine replacement therapy (NRT)*) on a scale from 1 (not harmful at all) over 3 (neutral) to 5 (very harmful). Finally, we asked all vapers if their health had changed since they started using e-cigs (yes–no question) and if so, exactly what had changed with regards to their health (open-ended question). To close, we asked participants to indicate how much they agreed with several statements on a scale from 1 (totally disagree) over 3 (neutral) to 5 (totally agree); each statement started with the phrase *“Due to using the electronic cigarette….*” and was followed by several potential benefits/improvements (such as quitting smoking, improvements on different health aspects, mood modification, etc.—for the full list, see Table 5).

### 2.3. Procedure

When participants had finished placing their order on the website (www.e-cig4u.nl), they were referred to the online questionnaire through a Qualtrics link. First, they received a short introduction to our study and an e-mail address in case of any questions. When they gave their consent to participate, the questions were presented as described and in the order mentioned in the Measures section. As a reward, one voucher of 25 € to spend in the web shop was raffled off among the participants. The study was conducted in accordance with the Declaration of Helsinki, and the protocol was approved by the Ethics Committee of Thomas More Mechelen-Antwerp University College.

## 3. Results

### 3.1. Statistical Analyses

Proportions and averages (and *SD*s) were computed using SPSS, version 24.0 (IBM, Armonk, NY, USA) [12]. All reported confidence intervals are lower and upper limits of Agresti-Coull confidence intervals of binomial proportions [13].

### 3.2. Smoking History and Smoking Status

Almost everyone in our sample (*n* = 201 out of 203) had ever been a smoker of tobacco cigarettes (see Table 2 for more details on smoking history and current smoking status). On average, those participants were 15 years and 3 months when they had started smoking and they had smoked for almost 30 years. A great majority had made at least one quit attempt in the past, with on average 4.7 quit attempts and the longest lasting period of smoking abstinence averaging around almost two years (for those ever having tried to quit).

Most of those who had made at least one quit attempt in the past (*n* = 166) reported that they had tried quitting smoking just on willpower, but only one-third reported this method (once) having been effective. Several different smoking cessation aids had been used by 1/10 to 1/2 of this sample, but these participants (who at least made one quit attempt in the past) reported these methods to have been not effective at all (see Table 2). Some of these participants also reported having used the e-cigarette as a smoking cessation aid and rated this as the most effective method (see Table 2). Less than one-fifth of the total sample was still smoking (see Table 2); those were rather low cigarette-dependent, and expressed highly variable degrees of motivation to quit (see Table 2).

Smokers reported experiencing several complaints due to smoking: mostly having a poor physical condition, worrying about their health, and cough tendencies. For an overview of all disadvantages with the distributions of the smokers’ responses, we refer to Table 3.

### 3.3. Vaping Status

Almost everyone (*n* = 195 out of 203) who participated had already tried an e-cig (96.1%, 95% CI 0.92, 0.98), and of those who had ever tried, 97.4% (95% CI 0.94, 0.99) were regular vapers. Vapers were using the e-cig for more than two years (*M* = 27.48 months; *SD* = 16.28), using e-liquid with an average nicotine concentration of 9.72 mg/mL (*SD* = 5.38). The propylene glycol/vegetable glycerine ratio of the base liquid was not assessed nor spontaneously mentioned by the participants, and no one indicated the use of marijuana/THC or any other inhalable drug as an additive. On average, regular vapers used 19.50 mL e-liquid per week (*SD* = 11.97), and used their e-cig 25 times per day (*SD* = 25.43), taking six puffs (*SD* = 4.51) per occasion they used the e-cig. Vapers indicated that they were/are mainly using e-cigs to quit smoking (73.3%, 95% CI 0.67, 0.79), because it is healthier than smoking (57.4%, 95% CI 0.50, 0.64), because of financial reasons (27.7%, 95% CI 0.22, 0.34), to decrease their tobacco cigarette consumption (16.9%, 95% CI 0.12, 0.23), because smoking is prohibited in several contexts (15.9%, 95% CI 0.11, 0.22), and out of curiosity (14.4%, 95% CI 0.10, 0.20).

Forty-nine different (brand) flavors were mentioned, and most vapers (94%, 95% CI 0.89, 0.97) had one favorite flavor—the most favorite ones being tobacco (55.3%, 95% CI 0.48, 0.63), fruit flavors such as apple, strawberry, raspberry (14.2%, 95% CI 0.10, 0.20), and mint (11.2%, 95% CI 0.07, 0.17). Because of the generality and/or incompleteness of the answers, it was not possible to get a detailed picture of the specific type of e-cigs each vaper used, but in summary most of them were using open system second-generation (for example Joyetech eGo-C or Kanger E-vod starter kits) or variable wattage third-generation devices (for example Eleaf iStick 20 W battery with Aspire Nautilus mini clearomizer), purchased from reputable sources and without modifications.

### 3.4. Perceived Harmfulness

When participants were presented with the following four options to choose between as to what they perceived as most harmful to their health: “*tobacco cigarettes*”, “*e-cigs*”, “*they are both equally harmful to my health*”, or “*they are both not harmful to my health*”, almost everyone perceived tobacco cigarettes as most harmful (94.3%, 95% CI 0.90, 0.97), a few (5.7%, 95% CI 0.03, 0.10) indicated *e-cigs and tobacco cigarettes were equally harmful*, nobody indicated *e-cigs as most harmful* nor chose the option *they are both not harmful to my health*. We also informed about the perceived harmfulness of smoking, vaping, smoking cessation medications, and NRT (see Table 4). The results (*n* = 188 out of 203 answered this question) clearly showed that almost everyone (97.3%) thought that smoking is harmful to very harmful. Vaping was considered not harmful at all to not harmful by the majority (52.6%); however, one-fifth of the participants perceived vaping to be harmful. Harm perception of vaping was similar compared to how participants rated the harmfulness of classic NRT and cessation medications.

Vapers were also asked about the disadvantages of both vaping and smoking. Almost all vapers (95%, 95% CI 0.90, 0.97) mentioned at least one of an extensive list of disadvantages due to smoking, mainly that smoking “contains chemicals”, “is carcinogenic”, “causes coughing” and “causes poor health”. The majority of vapers (59%, 95% CI 0.52, 0.65) reported no disadvantages about vaping, whereas those who did so almost exclusively reported some concerns about the nicotine in e-cigs and the addiction component of e-cigs.

### 3.5. Improvements in Health and Well-Being

A great majority (84.2%, 95% CI 0.78, 0.89) of vapers answered affirmatively to the question if their health had changed since they started using e-cigs. When asking what exactly had changed about their health, vapers reported solely positive things to this open question. The most frequently reported benefits were “improved breathing” (44.7%, 95% CI 0.37, 0.53), “less coughing” (34.9%, 95% CI 0.28, 0.43), “better physical health” (34.2%, 95% CI 0.27, 0.42), “feeling fitter” (17.1%, 95% CI 0.12, 0.24), “improved taste” (15.1%, 95% CI 0.10, 0.22 ), “improved smell” (11.1%, 95% CI 0.07, 0.17), “improved skin” (9.2%, 95% CI 0.05, 0.15), and “improved gums and teeth” (5.9%, 95% CI 0.03, 0.11).

More than 80% of the vapers agreed to totally agreed with the fact that their craving for tobacco cigarettes had decreased, that they could decrease their cigarette consumption, and could stop smoking due to using e-cigs (see Table 5). Most vapers also agreed that several aspects related to their health had improved due to using e-cigs, mainly having fresher breath, improved physical condition, breathing better, and improved smell and taste (see Table 5). Most vapers also agreed with the statement that they bothered bystanders less while vaping and that they could vape in more contexts than they could smoke (see Table 5). Half of the vapers (51.2%) agreed to totally agreed with the statement that they gained more pleasure from vaping than from smoking while only 9.5% indicated that vaping gave them less pleasure than smoking. Finally, perceptions of having “technical issues” with vaping were diverse.

## 4. Discussion

In this convenience sample of customers of an online vape shop in the Netherlands, we found that all were adults (18+), most had Dutch nationality, and about a quarter were Belgians. In some countries (like Belgium), the online sale of e-cig products is forbidden—purportedly to prevent the sale of such products to minors. At the time of the study, however, the law that forbids online sales in Belgium/to Belgians was not yet in place (law in force since 17 January 2017) [14], explaining the fact that almost a quarter of our sample had Belgian nationality. Although there was no formal age verification system to access the website or to place online orders, it seemed that in our sample this had not led to selling to minors, as the youngest customer who participated was 19 years old. Less than 1% (2 out of 203 participants) of the online customers had never smoked a tobacco cigarette before they started vaping—an observation which is in accordance with the findings obtained in the brick-and-mortar vape shop surveys [6,7], as well as with the findings of a recent survey by Phillips (*n* = about 20,000) among American vaping enthusiasts (members of the Consumer Advocates for Smoke-free Alternatives Association, CASAA) [5]. The results obtained in surveys with a less well-defined target population also point in the same direction. For example, in the first and largest “worldwide” online survey to-date (in more than 19,000 e-cigs consumers, 75% from Europe and 21% from the U.S.) carried out by Farsalinos and his colleagues [4], only 0.5% of the participants reported not being smokers at the time of e-cig use initiation. Finally, in the 2014 Eurobarometer (Eurobarometer 429) assessing smoking and vaping status in a representative sample (*n* = 27,801) of the population of the 28 European Union member states, current daily use of (nicotine containing) e-cigarettes was observed in 2.31% (2.13%) of current smokers and in 2.18% (2.13%) of former smokers, versus in no more than 0.08% (0.04%) of never smokers [15].

More than four out of five of our sample had made at least one attempt to quit smoking, with on average of almost five unsuccessful quit attempts. Most former smokers indicated that they tried to quit on their own, some used a form of NRT or smoking cessation medications (similar to other reports in the literature—e.g., [5]). Participants reported that these smoking cessation aids were not effective for them, in line with the low efficacy numbers of different smoking cessation aids found in the literature [16]. The results as to how many respondents indicated that they had used an e-cig as an (effective) smoking cessation tool are—at least at first sight—not clear-cut: Almost 50% of our sample indicated that they had tried it, but almost two thirds of our sample rated it as an effective smoking cessation tool. Since almost all participants were ex-smokers having completely switched to vaping, it is the authors’ best guess that those who did not report having used the e-cig as a smoking cessation aid, mainly had prior unsuccessful quit attempts in mind when answering this question, rather than reporting on the most recent one in which they had successfully switched to vaping. Alternatively, some of those not mentioning the e-cig as a smoking cessation aid may not categorize or label e-cigs as “smoking cessations aids”, or rather, they may have started vaping without the intent to quit smoking, but have quit accidentally.

Regarding changes in their smoking behavior, more than four of five agreed to totally agreed with the statements that they could quit smoking (81.1%), could decrease (including to zero cigarettes/day) their smoking consumption (85%), and that their craving for tobacco cigarettes had decreased due to vaping (92.8%). Again, these numbers are comparable to other, related research: 71.7% of the Canadian brick-and-mortar vape shop customers agreed that e-cigs are an effective way to quit smoking [6], whereas 87% indicated they quit smoking entirely after starting to use e-cigs in the CASAA survey by Phillips [5], and 81% of the 19,000 e-cig users in the study of Farsalinos and colleagues [4] reported complete substitution of smoking.

Those participants who were still smoking (dual users, 16.7% of the total sample) were low-dependent (*M_FTCD_* = 3.45) and consumed on average ten tobacco cigarettes per day. In comparison, 34% of the customers of the vape shop study of Tackett and colleagues [7] were dual users who smoked on average about seven cigarettes per day. In the CASAA study [5], only 5% of the vaping enthusiasts were dual users, and of those 34% smoked less than daily, 42% smoked one-to-five cigarettes per day, and 23% still smoked more than five cigarettes per day. Almost 20% of the 19,000 e-cigs consumers in the study of Farsalinos and colleagues [4] did not completely quit smoking, with one-third smoking less than daily and the remaining two-thirds smoking on average four cigarettes per day.

Of the customers who had just placed an order in the online vape shop, nearly everyone had tried an e-cig before. Almost all of those were regular vapers who had been using the e-cig for more than two years. Vapers indicated an average nicotine concentration of almost 10 mg/mL, and used open system second- or third-generation devices of good quality. More than 70% of our sample reported their favorite flavor being either tobacco or mint—traditional flavors that are associated with regular cigarettes. These numbers are higher than what is usually reported (e.g., [2]) and opposed to other samples indicating non-traditional flavors, such as fruity as most preferred (e.g., [5,7]).

The top three reasons that were most commonly agreed upon for using e-cigs were to quit smoking (73.3%), because e-cigs are healthier than smoking (57.4%), and because of financial reasons (27.7%). These reasons are quite commonly reported in other comparable studies [6,7]. Again, the results obtained in surveys with a less well-defined target population resulted in similar reported motivations for using e-cigs [1,2,3,4]. In addition to these (online) questionnaire studies, similar reasons for use were also reported by vapers who participated in qualitative focus group studies (e.g., [17]).

More than half (52.6%) of the participants in our study perceived vaping as *not that harmful* to *not harmful at all*, and although vaping was perceived as the least harmful among these products, still slightly more than 20% of the users believed vaping to be harmful. Given the availability of the other response alternatives (“not harmful at all”, “not that harmful”, and “neutral”), we interpret agreement with the “(very) harmful” statements as an endorsement of substantial health risk claims rather than as the rejection of a non-zero risk claim in about one out of five of this sample of vapers. These numbers are somewhat consistent with other reports. For example, in the vape shop studies, Tackett and colleagues [7] reported that participants perceived e-cigs as significantly less harmful than all other tobacco products, all NRT, and smoking cessation medications, whereas Volesky and colleagues [6] reported that 60.1% of their sample agreed or strongly agreed that e-cigs are harmless. In the above-mentioned study of Goniewicz and colleagues [3], most e-cig users perceived e-cigs as safer than tobacco cigarettes, but they did not think they were completely safe. Kozlowski, Homish, and Homish [18] reported that two-thirds of daily vapers believe that vaping is *a lot less dangerous* than smoking, whereas one-third think that vaping is *a little less dangerous*. Finally, in the online study by Farsalinos and his colleagues [4], 11% of the e-cigs users perceived e-cigs compared to tobacco as absolutely harmless and 88% as less harmless.

Most participants (84.2%) in our study agreed with health change statements since they had started vaping. Moreover, they only reported health benefits and no health disadvantages/complaints. This is consistent with earlier research in vapers in which consistently more health advantages due to e-cig use were reported than undesirable health effects (e.g., [2,4,7,19]), and in line with emerging evidence from clinical studies reporting (respiratory and cardiovascular) harm reversal in smokers having switched to vaping [20,21,22].

Our study provides new results on the usage, beliefs, and preferences of customers of an online vape shop in the Netherlands, but it also has some serious limitations. The first one concerns the generalizability of our findings to customers of other online vape shops than the one studied here. As could be expected based on the product portfolio, the online vape shop used in this study indeed attracted a population of regular open-system vapers. Nevertheless, we can only present it as a plausible hypothesis but not prove that it is representative for most other Dutch—and by extension European—vape shops. However, to the extent that the online customers of this vape shop would not substantially differ from the customers at other Dutch online vape shops, and knowing that there are about 30 other Dutch online vape shops catering to the “intermediate-level” vaper [23], it could be argued that our data potentially speak to on the order of several tens of thousands, perhaps more than a hundred thousand Dutch and Flemish vapers (estimate based on the number of online Dutch vape shops (30) multiplied by the number of registered online customers in the vape shop studied (23,000), but divided by a further unquantifiable factor greater than 1 to take into account that many vapers will shop at several online vape shops; that several customers may make only one purchase ever; and that several vape shops may have less or much less customers than the one studied here). Second, and more importantly, the sample studied suffered from substantial self-selection: it could be the case (and probably was the case) that mainly “the real enthusiast” (pro-vaping) customers participated in our study, whereas those feeling less positive about e-cigs or having been less successful with switching from smoking to vaping were more likely to decline participation. In that sense, the generalizability of our results to the target population—especially the findings concerning smoking quit rates and the experienced benefits of switching to vaping—is questionable indeed.

## 5. Conclusions

The study presented here is an extension of the North-American surveys of brick-and-mortar vape shop customers to those making purchases at an online vape shop. Apart from being the first survey of online vape shop customers, this study was also the first to ask detailed questions about Dutch (and Flemish) vapers’ usage, beliefs, and preferences. In conclusion, our findings are by and large similar to those obtained in the other vape shop studies [6,7], to the results of the CASAA study in about 20,000 American vaping enthusiasts [5], but also to information obtained in studies recruiting from less well-defined (broadly speaking North-American and/or European) populations using web-based convenience sampling or social media snowball sampling of vapers [1,2,3,4]. The latter suggests that in the few studies targeting a better-defined population of a geographically defined group of actual e-cig customers (like the brick-and-mortar vape shop studies, the CASAA study, and the one described here), a selection bias for having positive experiences with and being enthusiastic about vaping is probably as likely as in the studies with less well-defined populations. Nevertheless, the recurrently reported earlier unsuccessful smoking cessation attempts, using different aids such as NRT, and the overall agreement that vaping helps with quitting or reducing smoking in substantial proportions of respondents suffice to make the case that e-cig-based tobacco harm reduction (THR)—encouraging the substitution of low-risk alternatives—may provide a viable alternative for (at least some) smokers who cannot or do not want to cease all tobacco and/or nicotine consumption [24,25,26,27].

## Figures and Tables

**Table 1:** Sociodemographic characteristics (mean (*SD*) or %).

	*n*	*M* (*SD*) or %
**Demographic characteristics**		
Age (years)	203	46 (12.5)
Sex (men/women, %)	116/87	57.1/42.9
**Nationality (%)**		
Dutch	149	73.4
Belgian	46	22.7
British	3	1.5
American	1	0.5
Polish	1	0.5
Greek	1	0.5
Ukrainian	1	0.5
Swedish	1	0.5
**Educational degree (%)**		
Primary/Secondary school	50	24.6
High school	55	27.1
Bachelor	68	34
Master	20	9.9
Other (e.g., PhD)	10	4.9
**Employment status (%)**		
Blue-collar	96	47.3
Self-employed	23	11.3
White-collar	26	12.8
Retired	18	8.9
Looking for a job	17	8.4
Studying	8	3.9
Other (such as job in military)	15	7.4
**Net income per month (in €)**	177	2127 (1501)

**Table 2:** Smoking history and current smoking status (mean (*SD*) or % (95% CI)). FTCD: Fagerström Test for Cigarette Dependence.

	*n*	*M* or %	*SD* or 95% CI
**Smoking history**			
Ever smoked	201	99	0.96, 1.00
Age of smoking initiation	201	15.3	3
Years smoking	201	28.2	12.5
Made at least one quit attempt	166	82.6	0.77, 0.87
Number of quit attempts	166	4.7	8.4
Longest period of smoking abstinence (in months)	166	22.7	27
**Smoking cessation methods used/once being effective**	166		
Willpower	118/57	71.1/34.3	0.64, 0.77/0.28, 0.42
Nicotine patches	78/11	47/6.6	0.40, 0.55/0.04, 0.12
Nicotine gum	61/5	36.7/3.0	0.30, 0.44/0.01, 0.07
Smoking cessation medication	35/13	21.1/7.8	0.16, 0.28/0.05, 0.13
Nicotine tablets	21/2	12.7/1.2	0.08, 0.19/0.00, 0.02
E-cigarette	79/104	47.6/62.7	0.40, 0.55/0.55, 0.70
**Characteristics of current smokers**			
Smokers	34	16.7	0.12, 0.23
Cigarettes smoked per day	34	10.4	8.0
FTCD	34	3.5	2.6
Motivation to quit	34		
Not wanting to quit		32.4	0.19, 0.49
Thinking about quitting, but not within the next six months		20.6	0.10, 0.37
Thinking about quitting, within the next six months		17.6	0.08, 0.34
Thinking about quitting, within the next month		14.7	0.06, 0.31
Wanting to quit immediately		14.7	0.06, 0.31

**Table 3:** Distributions of responses (in %) of current smokers (*n* = 34) for several experienced disadvantages (on a scale from *never* to *always*) due to smoking.

	*Never*	*Seldom*	*Sometimes*	*Often*	*Always*
Poor physical condition	11.8	17.6	47.1	17.6	5.9
Worrying about my health	26.5	14.7	35.3	17.6	5.9
Cough tendencies	17.6	20.6	47.1	14.7	0
Unpleasant odors	23.5	23.5	29.4	23.5	0
Dry mouth	29.4	20.6	26.5	23.5	0
Bad smell	29.4	23.5	26.5	20.6	0
Dry throat	23.5	29.4	32.4	14.7	0
Bad taste	26.5	29.4	35.3	8.8	0
Breathing difficulties	26.5	35.3	32.4	5.9	0
Sleeping problems	38.2	29.4	11.8	20.6	0
Bad taste upon inhalation	44.1	17.6	29.4	8.8	0
Throat ache	38.2	38.2	14.7	8.8	0
Increased heart rate or palpitations	35.3	47.1	11.8	5.9	0
Head ache	35.3	47.1	11.8	5.9	0
Unpleasant sensation upon inhalation	50.0	23.5	20.6	5.9	0
Increased weight	55.9	23.5	8.8	8.8	2.9

**Table 4:** Distributions of responses (in %) of perceived harmfulness (on a scale from *not harmful at all* to *very harmful*) for smoking, vaping, smoking cessation medications, and nicotine replacement therapy (NRT).

	*Not Harmful at All*	*Not That Harmful*	*Neutral*	*Harmful*	*Very Harmful*
Smoking	1.1	0.5	1.1	25	72.3
Vaping	12.2	40.4	25.5	20.2	1.6
Smoking cessation medications	4.8	22.9	46.8	18.6	6.9
NRT	4.8	32.4	34.6	24.5	3.7

**Table 5:** Distributions of responses (in %) by current vapers (*n* = 195) on a list of several possible improvements due to using e-cigs (on a scale from *totally disagree* to *totally agree*).

	*Totally Disagree*	*Disagree*	*Neutral*	*Agree*	*Totally Agree*
My craving for a tobacco cigarette has decreased	1.7	2.8	2.8	16.7	76.1
I could decrease my smoking consumption	2.8	1.7	10.6	16.1	68.9
I could quit smoking	2.2	4.4	12.2	13.3	67.8
I have fresher breath	0.6	0.6	9.4	17.8	71.7
My physical condition and health has improved	1.1	2.2	13.3	22.8	60.6
I can breathe better	0	3.9	20.6	26.1	49.4
I have improved smell	0.6	2.2	30.6	27.2	39.4
I have improved taste	0.6	2.2	28.9	30	38.3
My appetite has improved	0	9.4	48.9	19.4	22.2
My quality of sleep has improved	1.7	10.6	61.1	8.3	18.3
I am in a better mood	2.8	8.9	61.1	11.7	15.6
I bother bystanders less	1.7	0	8.3	25.6	64.4
I can vape in more contexts	5	6.7	11.1	24.4	52.8
I gain more pleasure from vaping than from smoking	0.6	8.9	32.8	18.9	38.9
I have more technical issues	8.3	25.6	28.3	26.7	11.1
